# Pro-Inflammatory Versus Anti-Inflammatory Effects of Dendrimers: The Two Faces of Immuno-Modulatory Nanoparticles

**DOI:** 10.3390/nano7090251

**Published:** 2017-09-01

**Authors:** Séverine Fruchon, Rémy Poupot

**Affiliations:** 1 INSERM, U1043, Centre de Physiopathologie de Toulouse-Purpan, Université de Toulouse, F-31300 Toulouse, France; severine.fruchon@inserm.fr; 2 CNRS, U5282, F-31300 Toulouse, France

**Keywords:** dendrimers, nanoparticles, inflammation, immuno-safety

## Abstract

Dendrimers are soft matter, hyperbranched, and multivalent nanoparticles whose synthesis theoretically affords monodisperse compounds. They are built from a core on which one or several successive series of branches are engrafted in an arborescent way. At the end of the synthesis, the tunable addition of surface groups gives birth to multivalent nano-objects which are generally intended for a specific use. For these reasons, dendrimers have received a lot of attention from biomedical researchers. In particular, some of us have demonstrated that dendrimers can be intrinsically drug-candidate for the treatment of inflammatory disorders, amongst others, using relevant preclinical animal models. These anti-inflammatory dendrimers are innovative in the pharmaceutical field. More recently, it has appeared that some dendrimers (even among those which have been described as anti-inflammatory) can promote inflammatory responses in non-diseased animals. The main corpus of this concise review is focused on the reports which describe anti-inflammatory properties of dendrimers in vivo, following which we review the few recent articles that show pro-inflammatory effects of our favorite molecules, to finally discuss this duality in immuno-modulation which has to be taken into account for the preclinical and clinical developments of dendrimers.

## 1. Introduction

**Figure 1:**
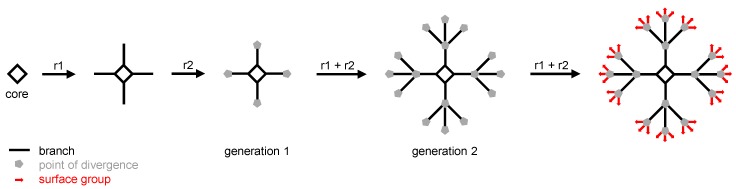
Schematic synthesis of a generation 2 (two series of branches) dendrimer using a tetravalent core (i.e., four branches in the first series) and trivalent points of divergence (i.e., 12 branches in the second series, and 36 surface groups). r1 and r2 are the reactions which are iterated to obtain the final dendrimer.

Since the first suggestion that perpetual synthetic branching should afford theoretically endless arborescent molecules, and after the pioneering syntheses of “octopus” [1] and “cascade” [2] molecules (called “dendrimers” later on [3]) by the group of F. Vögtle (to whom we dedicate this review), biologists and physicians have paid much attention to dendrimers. Dendrimers are built from a multivalent core on which successive series of branches are grafted thanks to a repeated sequence of quantitative reactions. At the end of each branch, a point of divergence is added which enables arborescence to be implemented at the next series of branches. Generally, the number of branches of a given series is twice or three times the number of branches of the previous series. The total number of series of branches determines the generation of the dendrimer: if it has one series of branches, it is a first generation (or generation 1) dendrimer; if it has two series of branches, it is a second generation (or generation 2) dendrimer, and so on ad libitum (Figure 1). Once the desired generation is obtained, the last step of the synthesis consists in the addition of the surface groups which will afford the outer shell of the dendrimer. As of today, the highest generation which has been reached is the thirteenth, affording large dendrimers (e.g., a molecular mass of 8.4 MDa and a size around 30 nm) matching the size of small viruses [4].

The following points summarize the main features of dendrimers that make them attractive nano-objects in the biomedical field:

(1) Due to their sequential process of synthesis, dendrimers have perfectly defined structures and molecular weights. Indeed, this important point distinguishes dendrimers from linear polymers (including synthetic glycosaminoglycans such as heparins and heparan sulfates) which are available in heterogeneous preparations. This is a real bottleneck for the advent of polymers as biomedical agents. On the contrary, provided an accurate stoichiometry is observed at each step of their synthesis, dendrimers are synthesized as isomolecular batches. This is a key point for the fate of dendrimers as new therapeutic and diagnostic tools in regard to regulation requirements;

(2) Their supramolecular properties are strongly involved in their uses: (i) supramolecular interactions with guest molecules (as cargos) inside the dendrimer; (ii) supramolecular interactions at the periphery of the dendrimer with substrates, and molecular or cellular targets to create nano-devices (examples are given below);

(3) Their nanometric size and globular shape are comparable to those of biomolecules (nucleic acids and proteins, respectively), and supramolecular biostructures (biological membranes and viruses). Of note, polysaccharides (such as heparins and heparan sulfates) are macro-biomolecules whose structures and bioactivities have inspired dendritic bioactive structures, as mentioned in paragraphs 2.1 and 2.4. One can assume that the size of a first generation dendrimer is 2 or 3 nm, and that 1 nm is gained with each supplemental generation. Therefore, dendrimers undoubtedly pertain to the “nanoworld”. Their structural and supramolecular characteristics make dendrimers perfect biomimics and carriers of biomolecules;

(4) Their multivalency enables polyvalent interactions with biotargets. The majority of biological molecular interactions occur through polyvalent bindings [5]. Natural multivalent ligands (with multiple receptor binding sites) or multivalent engineered nano-devices interact through polyvalent interactions with their partner receptors. The strength of these polyvalent interactions is called “avidity”, and is much higher than the sum of the single “affinities”. Typically, this is the case of carbohydrates which have weak binding affinities with receptor proteins. Their polymerization as natural or synthetic oligo- and polysaccharides strongly enhances the binding affinity with their cognate receptors [6]. Thus, from monovalent to polyvalent ligands, there is a strong enhancement in the intensity and duration of the stimulating signal which is delivered to a cell through a ligand-receptor interaction. This is called the “cooperative binding effect”; it has been thoroughly theorized by Mammen et al. [7], and it has been highlighted regarding dendrimers [8]. Therefore, dendrimers are perfect nanoparticles to enable polyvalent interactions involving ligands which are originally monovalent and, thus, to alter a biological process [9].

The advent of “nanotechnologies” in mass market products (food, cosmetics to name a few) and in the medical field has generated many discussions regarding both their enormous technological and economic potentials, and the specific environmental and health risks related to nanoparticle exposure [10]. Due to their small size, the properties of nanoparticles (including dendrimers) not only differ from raw, non-particulate material of the same composition, but also show different interaction patterns with the living organisms [9]. A risk assessment for raw, non-particulate materials is therefore not sufficient to characterize the same material in nanoparticle forms [11]. The implications of the special properties of nanoparticles have not yet been fully taken into account by regulators: e.g., size effects are not addressed in the framework of the new European chemicals policy REACH (Registration, Evaluation, Authorization and restriction of CHemicals). Nanoparticles raise a number of safety and regulatory issues that governments are now trying to tackle. In 2006, the FDA (Food and Drug Administration) established the Nanotechnology Task Force to address ways to evaluate nano-products. Since 2009, the European (EMA, European Medicines Agency), American (FDA), and Japanese (PMDA, Pharmaceuticals and Medical Devices Agency) agencies have worked together to achieve common perspectives for the development of nano-products. Nevertheless, at the international level, the development and the manufacturing of nano-products are still regulated by the same ICH (International Council for Harmonisation) guidelines as classical drugs (Q8 for Pharmaceutical development, Q9 for Quality Risk Management, and Q10 for Pharmaceutical Quality System). The regulatory agencies currently use pre-existing regulatory standards and statutes to regulate nanomedicines [12,13,14]. They are fighting to accumulate data and establish testing criteria, and agree that further basic research is necessary [14,15,16]. Studies on (i) the genotoxic, immunotoxic, hemolytic, carcinogenic, and teratogenic effects of the bioaccumulation and biopersistence of nanoparticles in the human body; and (ii) the ecotoxicity of nanoparticles (linked to their manufacturing and potential use in animal health) need a strong involvement of stakeholders, including authorities, industrials, and researchers, to go far beyond the current state-of-the-art.

Like all nanoparticles, dendrimers have undergone both reluctance and expectations at the same time. This is due to the fact that, over the years, dendrimers have found many fields of application [17]. In particular in the biomedical field, dendrimers are exploited as contrast imaging agents, diagnostic nano-tools, and drug carriers [18]. Indeed, an abundant and still ongoing literature, previously reviewed in Reference [19], shows how polycationic dendrimers are efficient as transfecting agents, bearing DNA at their periphery. More recently, polycationic dendrimers emerged as promising non-viral supramolecular nano-devices to carry and deliver small interfering RNA (SiRNA) in interference-based therapies [20]. Finally, dendrimer conjugates, either covalent or encapsulated, have shown pre-eminent features to enhance water solubility, to increase half-life, to improve cell targeting and uptake, or to decrease potential toxicity of drugs [21]. Besides, some dendrimers are promising drug candidates by themselves, in cancer, infections, and inflammation [22,23]. In particular, the assessment of the therapeutic efficacy of several families of dendrimers in recognized preclinical models of inflammatory disorders has generated reports in international journals with a broad audience, attesting the interest of the scientific community.

The current mini-review will start by focusing on articles that have demonstrated the intrinsic anti-inflammatory efficacy of dendrimers in vivo, in animal models of inflammatory diseases. Recently, at least two reports have mentioned pro-inflammatory effects of dendrimers in non-diseased animals. These two faces of immuno-modulatory dendrimers will be discussed, in light of further regulatory preclinical and clinical development. 

## 2. Review of Articles Dealing with Intrinsically Anti-Inflammatory Dendrimers

### 2.1. PolyAMidoAMine (PAMAM) Dendrimers

The first report assessing the therapeutic anti-inflammatory properties of dendrimers in vivo was published in 2004 [24]. Therein, two PAMAM dendrimers were tested in combination. One dendrimer is a generation 3.5 PAMAM ended by 64 carboxylic acid groups, nine of which had been conjugated to glucosamine residues (PAMAM-DG). This dendrimer has specific anti-inflammatory properties targeting human monocyte-derived macrophages and immature monocyte-derived dendritic cells activated with bacterial lipopolysaccharides (LPS). The dendrimer inhibits the release of pro-inflammatory cytokines by LPS-activated immune cells. The presence of the glucosamine residues is mandatory for the activity, as the carboxylated PAMAM skeleton alone was not active in the experimental setting. The second dendrimer is based on the same skeleton as the first, but is substituted with nine glucosamine-6-sulfate residues. This one has an anti-angiogenic activity in vitro. Based on these in vitro assessments, the authors administered a combination of the two dendrimers via subconjunctival and intraperitoneal injections in rabbits which had undergone experimental glaucoma filtration surgery. The readout was an improvement in wound healing of the surgery with a decrease of hypercellular scar tissue at day 30 after surgery. The combination of anti-inflammatory and anti-angiogenic dendrimers increases the long-term success of the surgery from 30% to 80% (i.e., a significant reduction of scar tissue). The anti-angiogenic activity of the PAMAM dendrimer decorated with nine glucosamine-6-sulfate residues is reminiscent of the same activity whose long heparan sulfate polymers are endowed.

### 2.2. From PAMAM to PolypropylETherIMine (PETIM) Dendrimers

Later on, the same PAMAM-DG dendrimer was proven to be efficient in preventing acute gut wall damage induced by severe inflammatory infectious diarrhea induced by *Shigella* [25]. When added directly in the infected ileum of rabbits, the PAMAM-DG dendrimer was able to reduce the copies of mRNA of interleukin (IL)-6, IL-8, and Tumor Necrosis Factor (TNF)-α (which are typical pro-inflammatory mediators), and to increase the number of copies of mRNA of IL-10 (the paradigm of anti-inflammatory cytokines) in the Peyer’s patches of the gut of infected animals. The PAMAM-DG is a large size (13.6 kDa) dendrimer, whose synthesis is difficult to scale-up, which harms its development for clinical use. Therefore, the authors employed a downsizing strategy to afford a series of smaller but still active dendrimers. This study was rationally based on molecular modeling approaches by the same group of authors. After having performed molecular dynamics simulations of the PAMAM-DG dendrimer [26], they showed that it is in fact an antagonist of the TLR4-MD-2-LPS complex, which therefore inhibits the pro-inflammatory effect of LPS on myeloid cells such as monocytes/macrophages [27,28]. This in silico-driven strategy afforded a new set of dendrimers, potentially bioactive, but with a reduced size (the smaller being a generation 3 dendrimer with a molecular weight of 3.3 kDa), and easier to synthesize: the PolypropylETherIMine (PETIM) dendrimers. When tested in the same animal model of severe inflammatory infectious diarrhea, the PETIM dendrimer was as efficient as the PAMAM-DG, although it was not able to increase the number of copies of mRNA of IL-10.

Finally, the 3.3 kDa PETIM dendrimer was evaluated in a non-human primate model of severe bacterial diarrhea. When given orally, the molecule is able to control the “cytokine storm” (i.e., the explosive increase of pro-inflammatory cytokines) induced by the gut infection by *Shigella* [29].

### 2.3. To Finish with PAMAM Dendrimers

In 2009, a series of PAMAM dendrimers intended to be conjugated with drugs were found to have intrinsically anti-inflammatory properties [30]. This has been assessed in three different rat models of inflammatory disorders: (i) the acute model of carrageenan-induced paw edema; (ii) the subacute cotton pellet model; and (iii) the chronic model of adjuvant-induced arthritis. The PAMAM dendrimers that have been tested are a generation 4.0 ended by –NH_2_ groups (G4–NH_2_), a generation 4.0 ended by –OH functions (G4–OH), and a generation 4.5 ended by –COOH groups (G4.5–CO_2_H). Of note, the latter corresponds to the dendrimer that had been derived from the glucosamine-conjugated dendrimer (PAMAM-DG) in Reference [24]. In the three animal models, the dendrimers were administered intraperitoneally. On average, G4–NH_2_ and G4–OH PAMAM dendrimers seem to have more or less the same activity, whereas the G4.5–CO_2_H dendrimer is much less active. From this point of view, this last result is consistent with that published in Reference [24], as it was claimed that glucosamine residues were mandatory for the anti-inflammatory activity of PAMAM-DG dendrimers.

The mechanism of action of G4–NH_2_ and G4–OH PAMAM dendrimers has been investigated in vitro. It was shown that they were able to inhibit the production of nitric oxide (NO, a pro-inflammatory mediator) by rat peritoneal macrophages activated by LPS. The G4.5–CO_2_H dendrimer is claimed to be less active than the two others. A second effect is the inhibition of the activity of cyclo-oxygenase 2 (COX-2) enzyme. COX-2 is activated in an inflammatory context and is a target of Non-Steroidal Anti-Inflammatory Drugs (NSAID).

More recently, another group assessed the anti-inflammatory efficacy of the same G4.5–CO_2_H PAMAM dendrimer and of a G5–OH PAMAM dendrimer in a mouse model of acute pancreatitis induced by caerulein [31]. The dendrimers were injected intravenously and both of them showed efficacy in decreasing the pathophysiological features of pancreatitis. The authors demonstrated in vitro that the G4.5–CO_2_H dendrimer is more active than the G5–OH in inhibiting the production of pro-inflammatory mediators by peritoneal macrophages activated by LPS.

### 2.4. PolyEthylene Oxide (PEO)/PolyEthylene Glycol (PEG) Dendrimers

So far, we have reviewed the anti-inflammatory properties displayed by dendrimers which target immune cells of the myeloid lineage such as monocyte-derived macrophages, immature monocyte-derived dendritic cells [24], and peritoneal macrophages [30,31]. Another way to alleviate inflammation is to inhibit the extravasation of leukocytes through the endothelial barrier to the sites of inflammation. Extravasation is initiated by selectin-induced leukocyte tethering and rolling on the endothelial surface. Selectins are glycoproteins of the lectin family, expressed both by leukocytes and endothelial cells. In return, leukocytes express O-glycosylated proteins that present carbohydrate epitopes consisting of sulfated derivatives of the tetrasaccharide sialyl Lewis X motif. Designing sulfated glyco-conjugate analogs of sialyl Lewis X as antagonist ligands for selectins is a promising track to develop anti-inflammatory drugs inhibiting extravasation.

To this aim, PolyEthylene Oxide (PEO) (or PolyEthylene Glycol, PEG) dendrimers (generation 1 and 2) ended by lactose groups on which hydroxyls can be naked, acetylated, or sulfated were synthesized [32]. The anti-inflammatory activity of these candidates was assessed in an acute inflammatory model induced in mice by thioglycollate injection into the peritoneal cavity. Whereas the PEO/PEG dendrimers of the first generation showed little activity, the sulfated PEO dendrimer of the second generation dramatically reduced the recruitment of neutrophils and macrophages. In this system, the dendritic scaffold takes advantage of a multivalent ligand presentation to have a degree of bioactivity similar to the natural heparan sulfate polymer, which is an antagonist of selectin ligands and inhibits extravasation.

Following the same track, others have designed dendritic polyglycerol sulfates (dPGS) as heparin analogs [33]. They have varied several structural parameters of the dendrimers (the size of the internal scaffold and the rate of sulfation). The efficacy of these dendrimers in inhibiting the extravasation of leukocytes has been proven in a mouse model of acute allergic contact dermatitis with typical symptoms: redness, ear swelling, edema, and cellular infiltration. Dendrimers were injected subcutaneously. The most active compound is the most multivalent, i.e., the one bearing the highest number of sulfate groups (61 in this case).

### 2.5. Phosphorus-Based Poly(PhosphorHydrazone) (PPH) Dendrimers

Phosphorus-based dendrimers are built from a hexa-functional cyclo-triphosphazene core on which series of branches bearing Poly(PhosphorHydrazone) (PPH) motifs can be grafted. When AzaBisPhosphonate (ABP) surface groups are added at the outer series of branches, we obtain anti-inflammatory dendrimers. In this series, we have shown that the generation 1 ABP dendrimer is the most active (molecular weight 5.8 kDa). It targets human monocytes in vitro. Indeed, the dendrimer is specifically recognized and internalized by these cells [34], and it activates them towards an anti-inflammatory response by promoting the increase of the transcription of anti-inflammatory mediators [35]. When other anionic surface groups such as azabiscarboxylates or azabisulfonates are added instead of ABP, the molecules are no more active [36]. Recently, we compared the structure and the anti-inflammatory activity of different types of dendrimers (i.e., built on different types of internal skeletons), all of them bearing ABP groups at their surface. We showed that the three-dimensional structure of the dendrimer is key. To be bioactive, the dendrimer must present its surface groups in a directional way, therefore optimizing the benefits of multivalency [37]. The generation 1 ABP dendrimer, which is the most active in vitro towards primary human immune cells, was challenged in mouse models of inflammatory diseases.

At first we used a mouse model of experimental arthritis which is relevant to Rheumatoid Arthritis, a chronic inflammatory disease of auto-immune origin. In this model there is a constitutive inflammatory context due to the absence of the antagonist to the receptor to IL-1 (a potent pro-inflammatory cytokine). In this model, the ABP dendrimer is active at 1 and 10 mg/kg/week via intravenous injection [38]. It also works via oral route (gastric gavage) [39]. By targeting inflammatory monocytes and switching them toward a benefic anti-inflammatory phenotype, the ABP dendrimer inhibits the secretion of pro-inflammatory mediators and enhances the production of IL-10. Thus, it controls the inflammation at the systemic and articular levels. It also decreases the secretion of Matrix MetalloProteases (MMP), preventing the degradation of cartilage; and it inhibits the differentiation of monocytes in osteoclasts, the giant multinuclear cells responsible for the resorption of bones.

In another mouse model, we showed that the same molecule is efficacious in controlling the onset and development of Experimental Auto-immune Encephalomyelitis (EAE, a preclinical model of Multiple Sclerosis) [40]. In this model, we showed that the ABP dendrimer controls and inhibits inflammation in at least two different ways. It mitigates the capability of antigen-presenting cells (mainly monocytes and dendritic cells), and promotes the differentiation of IL-10-producing CD4^+^ T lymphocytes. These two properties highlighted in the EAE mouse model are reminiscent of what we observed in vitro in primary human immune cells [41,42].

Finally, the ABP dendrimer is also effective in controlling uveitis in a rabbit model after loco-regional (intravitreal) administration [43]. Once again, it is active by promoting the increase of IL-10, as detected in the serum of the treated animals.

Another series of PPH dendrimers was shown to be anti-inflammatory in a mouse model of acute lung inflammation [44]. They are built on the same scaffold as ABP dendrimers, they are generation 1 to 4 molecules, and they bear oligomannoside surface groups (mannodendrimers). They are intended to mimic the mannose-capped lipoarabinomannan of *Mycobacterium tuberculosis*. This supramolecular natural structure binds to Dendritic Cell-Specific Icam-3 (IntraCellular Adhesion Molecule 3)-Grabbing Nonintegrin (DC-SIGN) on dendritic cells and inhibits the release of pro-inflammatory cytokines. Among the set of mannodendrimers that have been synthesized, the most efficacious are the largest (generation 3 and 4), i.e., with molecular weights of 48 and 77 kDa. However, this strongly impairs their preclinical and clinical developments.

## 3. Immunological Breaking News: Pro-Inflammatory Dendrimers Do Exist

For the first time in 2016, it was claimed that PAMAM dendrimers could have pro-inflammatory effects in vivo [45]. Generation 0 to generation 3 PAMAM dendrimers terminated by –NH_2_ functions were tested in the mouse air pouch model. When injected in the air pouch, these dendrimers enhance the infiltration of leukocytes, the vast majority of them being neutrophils which are major cellular players of inflammation. Of note, the increase is statistically significant for the generation 3 dendrimer only. As a consequence, there is an increase of the level of pro-inflammatory mediators in the exudates taken from these air pouches.

These results can be brought together with an observation we published in 2015 [46]. In an unprecedented study, we administered the anti-inflammatory ABP dendrimer in healthy non-human primates to detect early toxicity and potential immunosuppression. The dendrimer was administered intravenously four times at 10 mg/kg with one-week intervals. Clinical observation was performed twice a day during the assay, and biochemical and hematological parameters were measured periodically before and after each injection of the dendrimer. Interestingly, we observed that the level of C-reactive protein and aspartate- and alanine-amino-transferases (ASAT and ALAT, enzymes that are upregulated by the liver in an inflammatory context) were increased after the first injection. These parameters came back at their normal level within two or three days, and there was no cumulative effect at the following injections. These events clearly indicate that the ABP dendrimer induced a mild inflammatory boost at the first injection. This unexpected boost had no deleterious effect on the monkeys, as confirmed by the analysis of the main thoracic and abdominal organs (the liver in particular) at necropsy.

## 4. Discussion

Since 2004, many families of dendrimers, ended with a variety of surface groups, have shown intrinsic anti-inflammatory properties validated in relevant animal models of inflammatory disorders. The diversity of these animal models as well as the different routes of administration that have been used may explain why different results are obtained with very close compounds, sometimes even with the same molecule. Surprisingly, recent reports showed that some of these dendrimers can have either anti-inflammatory or pro-inflammatory effects. Of course, this duality is observed in different in vivo models. In each case, the pro-inflammatory boost generated by the concerned dendrimers was observed in non-diseased models: the air pouch mouse model for PAMAM dendrimers [45], and healthy non-human primates for the ABP phosphorus-based dendrimer [46]. One can hypothesize that these pro-inflammatory effects, if any, should have been masked in inflammatory animal models. Nevertheless, these observations raise the issue of long-term use of therapeutic dendrimers to treat chronic affections in humans. On the whole, using dendrimer-based—as well as other nanoparticle-based—diagnostics or therapeutics in humans raises the issue of their long-term bio-accumulation, general systemic toxicity, genotoxicity, and immunotoxicity [47,48].
